# Tailored Prediction Model of Survival after Liver Transplantation for Hepatocellular Carcinoma

**DOI:** 10.3390/jcm10132869

**Published:** 2021-06-28

**Authors:** Indah Jamtani, Kwang-Woong Lee, Yunhee Choi, YoungRok Choi, Jeong-Moo Lee, Eui-Soo Han, Kwangpyo Hong, Gyu-Seong Choi, Jong Man Kim, Nam-Joon Yi, Suk Kyun Hong, Jeik Byun, Su Young Hong, Sanggyeun Suh, Jae-Won Joh, Kyung-Suk Suh

**Affiliations:** 1 Department of Surgery, Seoul National University College of Medicine, Seoul 03080, Korea; indah.jamtani69@gmail.com (I.J.); choiyoungrok@gmail.com (Y.C.); lulu5050@naver.com (J.-M.L.); uishann@gmail.com (E.-S.H.); gigsfire@gmail.com (K.H.); gsleenj@hanmail.net (N.-J.Y.); nobel1210@naver.com (S.K.H.); jeikbyun@gmail.com (J.B.); sypurple@gmail.com (S.Y.H.); sgsuh@gmail.com (S.S.); kssuh2000@gmail.com (K.-S.S.); 2 Division of Medical Statistics, Medical Research Collaborating Center, Seoul National University College of Medicine, Seoul 03080, Korea; yhchoi@snuh.org; 3 Department of Surgery, Samsung Medical Center, Sungkyunkwan University School of Medicine, Seoul 06351, Korea; gyuseong.choi@samsung.com (G.-S.C.); jongman94@hanmail.net (J.M.K.); jwjoh@skku.edu (J.-W.J.)

**Keywords:** hepatocellular carcinoma, liver transplantation, survival, recurrence

## Abstract

This study aimed to create a tailored prediction model of hepatocellular carcinoma (HCC)-specific survival after transplantation based on pre-transplant parameters. Data collected from June 2006 to July 2018 were used as a derivation dataset and analyzed to create an HCC-specific survival prediction model by combining significant risk factors. Separate data were collected from January 2014 to June 2018 for validation. The prediction model was validated internally and externally. The data were divided into three groups based on risk scores derived from the hazard ratio. A combination of patient demographic, laboratory, radiological data, and tumor-specific characteristics that showed a good prediction of HCC-specific death at a specific time (t) were chosen. Internal and external validations with Uno’s C-index were 0.79 and 0.75 (95% confidence interval (CI) 0.65–0.86), respectively. The predicted survival after liver transplantation for HCC (SALT) at a time “t” was calculated using the formula: [1 − (HCC-specific death(t’))] × 100. The 5-year HCC-specific death and recurrence rates in the low-risk group were 2% and 5%; the intermediate-risk group was 12% and 14%, and in the high-risk group were 71% and 82%. Our HCC-specific survival predictor named “SALT calculator” could provide accurate information about expected survival tailored for patients undergoing transplantation for HCC.

## 1. Introduction

Hepatocellular carcinoma (HCC) is the prevailing primary liver cancer and the third leading cause of cancer-related deaths worldwide [1]. The estimated incidence is one million new cases annually worldwide [2]. Liver transplantation (LT) is an option for treating selected cases of HCC. The advantage of LT is that it removes the underlying diseased liver while eliminating the tumor [2,3,4]. The criteria for LT in HCC, especially for living donor liver transplant (LDLT), has been expanding in recent years, as the overall survival has been excellent. This poses new challenges since the recurrence rate has started to increase [1,2,4]. In the past two decades, there have been advancements in the selection criteria. In addition to tumor parameters (size and number of nodules), biomarkers, such as alpha-fetoprotein (AFP) and protein induced by vitamin K absence (PIVKA-II), and histopathologic characteristics were included. However, specific criteria focusing on tumor-specific survival post LT for HCC have been hard to establish [1,4,5]. Since recurrences after LT were considered to have a poor prognosis in previous studies, many protocols, and screening criteria were developed to accurately predict recurrence after LT instead of concentrating on survival despite tumor recurrence or tumor-specific survival [1,2,5].

Recently, owing to improvement in the tools for selection based on favorable tumor biology, incorporation of mammalian target of rapamycin inhibitors (mTORi), and aggressive local treatment after recurrence, there is a belief that tumor recurrence, however, a feared event, does not immediately mean poor survival outcomes [1,3,5]. Therefore, it was time to develop survival-oriented criteria. This study aimed to create a tailored pre-transplant model to predict HCC-specific survival after transplantation.

## 2. Methods

### 2.1. Patients

#### 2.1.1. Derivation Set

From June 2006 to July 2018, 739 adult patients underwent LT and had pathologically confirmed HCC at Seoul National University Hospital (SNUH), Korea. Among them, 578 cases with available 18F-fluorodeoxyglucose positron emission tomography (18F-FDG-PET) data were included in this study. All variables were collected from a prospectively recorded database for those with a minimal follow-up of 18 months (1.5 years).

#### 2.1.2. Validation Set

A total of 210 pathologically confirmed cases were collected from the Samsung Medical Center, Korea, between January 2014 and June 2018. This period was chosen since PIVKA-II and 18F-FDG-PET were in routine use and mTORi administration in advanced and recurrence cases, making the criteria of the validation set similar to the derivation set. Cases lacking PET imaging (*n* = 15) and critical laboratory values (*n* = 20) were excluded from the analysis. 

### 2.2. Demographic Characteristics and Definitions

Pre- and post-transplant data were prospectively recorded without modifications, including patient demographic, laboratory, and radiological data. Tumor morphological characteristics were collected from the pathological reports of explanted livers and not from preoperative radiological data, under the assumption that both reflected the same measurements. The largest tumor size was measured as the largest diameter of the largest tumor (mm), regardless of necrosis. Neutrophil-lymphocyte ratio (NLR) was calculated as the relative percentage of neutrophils over lymphocytes. PET positivity and degree of positivity is determined by visual analysis, reinforced by tumor liver standardized uptake value (SUV) ratio. Tumor liver ratio (TLR) is defined as the highest uptake of 18F-FDG in the region of interest (T_max_) over the highest uptake value of the normal liver (L_max_). Mild and strong hypermetabolic lesions were determined by visual analysis. We defined an iso-metabolic lesion as 18F-FDG-PET negative and a mild or strong hypermetabolic lesion as PET positive for clinical lenience. 

### 2.3. Pre-Transplant Evaluation

In addition to computed tomography (CT) and enhanced magnetic resonance imaging (MRI), 18F-FDG-PET was added to the pre-transplant HCC workup since mid-2006 in selected cases. However, it was not until 2007 that 18F-FDG-PET was routinely implemented. Therefore, patients with missing PET data were excluded from this study. Tumor markers, including AFP and PIVKAII, were also included for evaluation. Selection criteria for transplantation based on our standard clinical practice guidelines were expanded by including AFP, PIVKAII, and PET positivity instead of size and number alone. Patients with extrahepatic metastasis were contraindicated for our study. 

### 2.4. Post-Transplantation Management and Follow-Up

The post-transplantation immunosuppression regimen has been described previously [3,4,5,6]. Briefly, the triple combination regimen consisting of tacrolimus, mycophenolate mofetil (MMF), and steroids after basiliximab induction was used. mTORi was considered in selective patients, administered one month after LT when HCC was advanced and have a high risk of tumor recurrence, and given in every case of recurrence. 

Follow-up was performed once a week during the first month post-transplant, twice a month until three months, monthly during the first year, and once every 3 or 4 months in subsequent years. Serum AFP and PIVKAII levels were evaluated during every visit. Abdominopelvic CT or MRI, bone scan, and chest CT were performed every 3–6 months or in case of increasing AFP levels to check for HCC recurrence. The 18F-FDG-PET was performed when there was a high suspicion of recurrence, but the abdominal images were negative [7].

This study followed the ethical guidelines of the World Medical Association Declaration of Helsinki and was approved by the institutional review boards of both partaking centers: Seoul National University Hospital (IRB no. 2009-125-1159) and Samsung Medical Center (IRB no 2020-08-007).

### 2.5. Statistical Analyses

Continuous data were expressed as mean (standard deviation) or median (range) and tested using t-test or Wilcoxon rank-sum test depending on normality. Categorical data were expressed as numbers with percentages and tested using the chi-squared test. The cumulative incidence rate of HCC-specific death (HCCD) was estimated by accounting for the competing risk of non-cancer-specific death. A univariable and multivariable Fine and Gray competing risk regression model was used to predict HCC-specific mortality. Significant predictors with a significance level of 0.2 from the univariable analysis were included in the multivariable analysis. The linearity of a continuous predictor with log-hazard ratios of the cumulative incidence function was checked by categorizing the predictor and examining the coefficients for each category. Since AFP and PIVKAII violated the linearity assumption, log-transformation was performed. The assumption of proportionality for the hazard ratio was checked using the time-by-covariate interaction for each predictor. Only predictors with a significance level of 0.05 were included in the final model. Internal and external validations were performed. Internal validation was performed using bootstrapping with 1000 bootstrap datasets. As measures of predictive performance assessing calibration and discrimination, calibration plot, calibration slope, and Uno’s c-index were calculated. The cutoff value of the risk score (RS) in the three groups of the derivation set was determined to show significant survival differences after binning into groups with small ranges. The statistical analyses were performed using a combination of SPSS version 26 (IBM Corp., Armonk, NY, USA) and SAS^®^ version 9.4 (SAS Institute Inc., Cary, NC, USA). 

## 3. Results

### 3.1. Demographic and Clinical Characteristics

Baseline demographic and clinical characteristics are shown in Table 1. The mean age was 55.6 (±8.3) and 56.7 (±6.3) years, and there were 472 (81.7%) male patients in the derivation set and 153 (87.4%) male patients in the validation set. The p-values were 0.06 and 0.07 in the sets, respectively. The majority of patients underwent LDLT: 85.6% in the derivation set and 96% in the validation set. Hepatitis B was the most common underlying disease in both sets. 

**Figure 1:**
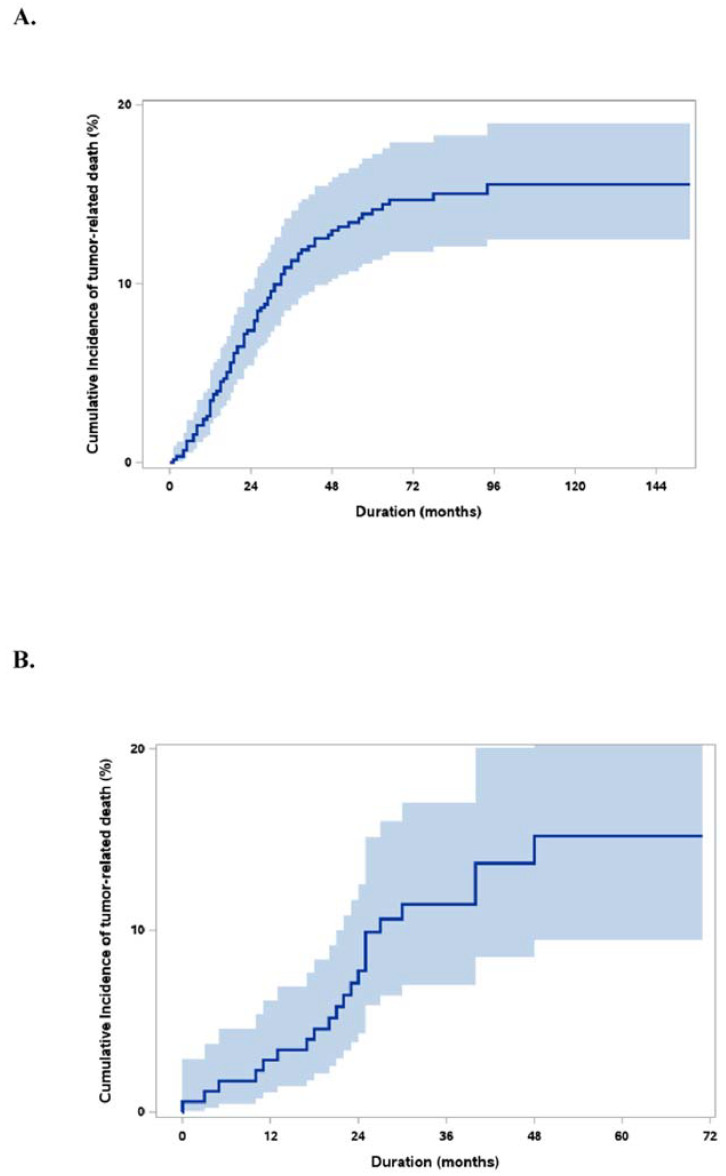
Cumulative incidence curve of HCCDs (95% CI). (**A**). Derivation set. (**B**). Validation set.

No differences were found between the sets in terms of PET positivity, AFP and PIVKAII levels, and the largest tumor size irrespective of viability. However, the median NLR ratio and model of end-stage liver disease scores were significantly higher in the derivation set than in the validation set. The median follow-up period was 65.5 (0–154) months in the derivation set and 34 (0–71) months in the validation set (Table 1). In this study, the 5-year overall survival was 80.3%. The cumulative incidence rates of HCC-specific death were 3% and 11% in the first and third years after LT in both sets, respectively. The 5-year HCC-specific death rate was 14% in the derivation set and 15% in the validation set (Figure 1).

### 3.2. Derivation of Tailored Survival Calculator

The Fine and Gray regression model was applied in the derivation set. In the multivariable analysis, six variables were identified as risk factors for HCC-related death: sex, ln (AFP+1), ln (PIVKAII), PET positivity, largest tumor size (mm), and NLR. While sex and PET positivity were categorical variables, other variables were continuous variables (Table 2). 

Based on this analysis, the cumulative incidence of HCC-specific death (HCCD) was calculated using the following equation: 

RS = 0.96 × if male + 0.01 × largest tumor size (mm) + 0.88 × if PET-positive + 0.21 ln (AFP+1) + 0.17 × ln (PIVKAII) + 0.02 × NLR.

The cumulative incidence rate of HCCD at a specific time “t” was calculated using the following equation:

[HCCD(t)] = 1−exp(−exp(RS)×Incidence Probability(IP)(t)), where IP(t) is the cumulative incidence at a time “t” with RS = 0. The IP(1-year), IP(3-year), and IP(5-year) were 0.001, 0.005, and 0.007, respectively. 

The predicted tumor related “Survival After Liver Transplantation” for HCC (SALT) at time “t” = [1-HCCD(t)] × 100.

### 3.3. Validation

**Figure 2:**
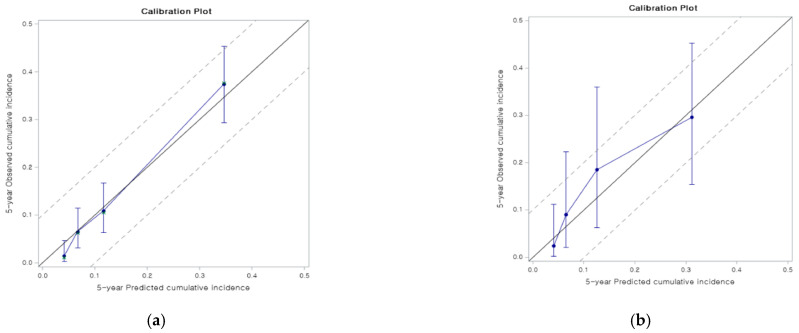
Internal and external validations of the developed model. The model shows reasonable discrimination with Uno’s c-index of calibration and slopes in internal (**a**) and external (**b**) validation.

The model shows reasonable discrimination with Uno’s c-index of calibration of 0.8 (95% confidence interval [CI] 0.75–0.86). The bootstrap-corrected Uno’s c-index was 0.79 for internal validation (Figure 2a) and Uno’s c-index for external validation was 0.75 (95% CI 0.65–0.86) (Figure 2b) (Table 3). The calibration slopes of 1 (95% CI 0.84–1.16, bootstrap-corrected slope 0.85) and the calibration plot in the internal validation showed a good correlation. Moreover, the model showed good calibration in the external validation: the calibration slope was 0.9 (95% CI 0.49–1.3) even though the model tended to slightly underestimate the cumulative incidence for patients whose cumulative incidence at five years was between 0.1 and 0.2 in the external validation. 

### 3.4. Groups by Risk Score

**Figure 3:**
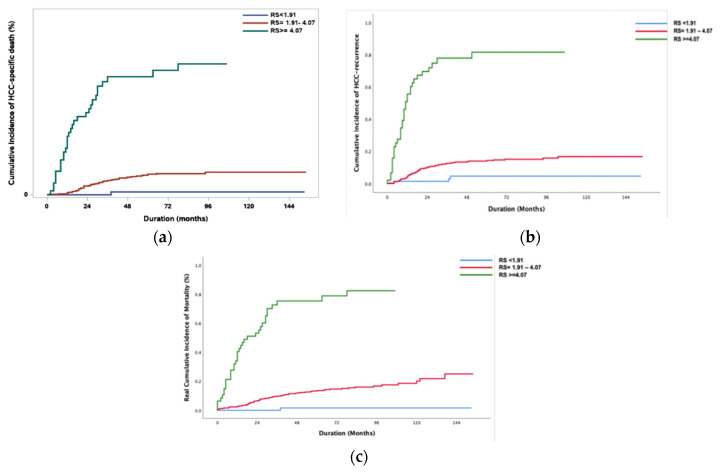
Different incidence rates categorized based on the range of risk scores (RS): (**a**) cumulative incidence rates of HCC-specific death accounting for competing risk of non-cancer-specific death. (**b**) Cumulative incidence of HCC recurrence. (**c**) Real cumulative incidence of mortality in the derivation set according to the risk score.

The RS was calculated in 554 patients in the derivation set (24 cases had missing NLR values), and they were divided into three groups. Group I was categorized as a low-risk group with RS ≤ 1.91 (*n* = 74); group II as an intermediate-risk group, RS 1.91–4.07 (*n* = 433); and Group III as a high-risk group, RS > 4.07 (*n* = 47). The cumulative incidence of HCCD and HCC recurrence was significantly different between the groups (*p* < 0.01) (Figure 3a,b). Group I showed 0% HCCD and HCC recurrence in the first and third years and a 2% 5-year HCCD and 5% HCC recurrence. The 5-year HCCD and HCC recurrence in Group II were 12% and 14%, respectively, and in Group III were as high as 71% and 82%, respectively (Table 4 and Table 5). The overall death, including non-HCC-related death between groups, is presented in Figure 3c and Table 6. The pattern of recurrence was analyzed (Table 7). A shorter recurrence-to-death period (tumor-bearing survival (TBS)) was observed in Group III than in Group II with a borderline significance (*p* = 0.06). However, no difference was noted between the groups in terms of the initial sites of recurrence. We also compared the recurrence and HCCD rates between LDLT and DDLT in each risk group. There was no significant difference in recurrence and HCCD rates between LDLT and DDLT in all risk groups; *p* = 0.41, *p* = 0.86, and *p* = 0.78 for recurrence, and *p* = 0.643, *p* = 0.29, and *p* = 0.53 for HCCD in Group I, II, and III.

## 4. Discussion

In recent years, more than 100 LTs have been performed annually, with approximately 60% of cases being performed for HCCs in our center [3,6]. Patient selection in our center is based on tumor biomarkers, including AFP and PIVKA-II levels and 18F-FDG PET positivity. Pre-LT serum PIVKA-II levels have been checked routinely since 2005, and the use of PET scan as a pre-LT screening tool was started in mid-2006 but was only routinely performed since 2007 [3,6,8,9]. All three positive biological factors in a patient is considered a relative contraindication regardless of Milan criteria. Administration of mTORi in patients with advanced HCC based on pre-LT biological factors and patients with recurrence started in 2005 [3,6]. This study enrolled patients from June 2006 until July 2018 to select a unique set with similar pre- and post-transplant management protocols and an adequate follow-up period.

Even with advanced improvements in selection criteria, the majority still focus on recurrence. However, tumor recurrence after transplantation does not always result in death. There have been several improvements to prolong survival even after recurrence following LT, such as (1) selection of patients with more favorable tumor biology, (2) early administration of mTORi in advanced and recurrence cases, and (3) early aggressive local management and combination of targeted chemotherapy for tumor recurrence [1,3]. In a previous report, we described long-term TBS as that >3 years after HCC recurrence in 13 recipients (14% of the whole patient cohort with recurrence) [3]. Therefore, it is time to focus on tailored HCC-specific survival or individualized survival benefit of LT over other treatments rather than recurrence-free survival (RFS) itself. However, to the best of our knowledge, no studies have focused on pre-transplant data to predict tumor-specific survival [5].

Determination of HCC-specific mortality continues to be challenging. Factors affecting survival in patients who undergo LT for HCC vary from patient performance status to risk of de novo cancer and accidents [5]. Thus, competing risk analysis is imperative for predicting HCC-related deaths alone in these settings. The six variables significant for HCCDs in our study were sex, PET positivity, AFP and PIVKA-II values, largest tumor size regardless of viability, and NLR. The combination of these variables was used to create an end model for tumor-specific survival prediction named the Survival After Liver Transplantation for HCC (SALT) calculator.

Tumor morphology, mainly tumor size, and number have been included as selection criteria from the very beginning. Following the disappointing results of LT in HCC cases in the late 1980s, Mazzaferro et al. developed the Milan criteria in which a solitary tumor < 5 cm in diameter, multiple tumors up to 3 cm, or the largest tumors with a size of 3 cm showed good survival outcomes post LT [10]. Another more recent criteria based on tumor morphology is the University of California, San Francisco (UCSF) criteria, in which a solitary tumor ≤ 6.5 cm; ≤ 3 tumors, none greater than 4.5 cm; and total tumor diameter not surpassing 8 cm were similarly reported to have excellent 5-year survival outcomes [11]. In our multivariable analysis, the size of the largest tumor regardless of viability, as a continuous variable, was indeed a significant risk factor for HCCD; the larger the size, the higher the risk of HCCD post-LT.

Tumor size data were collected from pathology reports using the total size (including the necrotic area) of the largest tumor on the specimen. Although these were post-transplant data, we assumed that the pre-transplant radiological tumor size would be the same as the pathological size. 

Previous studies had shown a 25–30% of inaccuracy rate of imaging diagnosis. Shah et al. (2006) reported a significant understaging with computed tomography (CT) and ultrasonography (US) diagnosis compared with explant pathology [12]. However, in a study comparing CT scan and MRI in HCC diagnosis in 2018, Wang et al. reported a higher sensitivity, specificity, and accuracy in MR, especially in small HCCs [13]. There is continuing progress in the radiologic diagnostic tools of HCC to derive complete and accurate morphological characters of the tumors. Therefore, we decided to use the explant tumor size to obtain a more reliable and precise prognostic prediction hoping that the radiologic diagnosis is more accurate in the near future.

Pre-LT treatment tumor responsiveness is indeed an important factor in assessing post-LT HCC recurrence and survival. Our institution has previously reported post-Transcatheter arterial chemoembolization (TACE) tumor responsiveness as a risk factor in port-LT recurrence: Park et al. (2014) reported that minimal (≤60%) post-TACE tumor responsiveness is a risk factor for post-LT HCC recurrence [7]. In this study, we used various types of tumor size variables including the size of viable portion of the largest tumor or the total size regardless of pre-LT treatment related necrosis in the multivariate analysis. Statistical significance was found in both variables. Although tumor necrosis was also an important factor, we decided to include total size regardless of necrosis of the largest tumor nodule in the final model considering clinical convenience as calculating tumor necrosis radiologically is not always accurate, or even feasible.

Pre-transplant serum AFP level has been frequently associated with tumor progression and recurrence and is an independent risk factor for patient survival after LT [5,9,14,15]. In our previous studies, AFP levels >400 ng/mL have shown significant survival risk, and when combined with 18F-FDG PET positivity, AFP level of 200 ng/mL showed significant risk [6,9,14]. Furthermore, serum PIVKA-II is an impelling factor for portal vein invasion, intrahepatic spread, and extrahepatic metastasis by promoting epithelial-mesenchymal transition [8,16,17,18]. Recently, a model to predict tumor recurrence after LDLT (MoRAL), which combined serum AFP and PIVKA-II, was developed in our center [8] While individual log (AFP+1) and PIVKA-II values were chosen in the SALT calculator, we combined these with other significant variables and tried various modifications (continuous vs. categorical, law value, square root conversion, logarithm conversion, etc.) to project survival better. The MoRAL score that used the square root of AFP and PIVKAII values was also statistically significant but less appropriate than log conversion. 

Tumor aggressiveness in HCC could be reflected by the high glucose metabolism of the tumor. Enhancement of glucose transporter-2 on the cell membrane and glycolytic enzymes, such as hexokinase-2, usually means the presence of an aggressive tumor that is growing rapidly. The 18F-FDG is an analog of glucose, and 2-deoxyglucose is an analog of 18F-FDG; thus, it can be transported by glucose transporters and phosphorylated into 18F-FDG-6-phosphate. This molecule becomes confined in cells, accumulates in proportion to the glucose metabolism, and is therefore highly concentrated in an aggressive viable tumor [9,19].

Varying degrees of PET-parameter cutoff values have been used in the literature, evaluating the predictive value of 18F-FDG PET in HCC patients with surgical resection ranging from 1.10 SUV to 6.36 TLR [20]. Our center also published a study reporting the cutoff value for 18F-FDG PET positivity, using the receiver operating characteristic (ROC) curve, was TLR (Tmax/Lmax) SUV ratio of 1.10 [9]. However, in this study, mild and strong hypermetabolic lesions were determined by visual analysis, reinforced by TLR SUV ratio. This simplified version provided a straightforward assessment and was practical in clinical settings. 

Cancer-associated inflammation is linked with outcomes in various malignancies. Hematological inflammatory markers, particularly NLR, were investigated enthusiastically as a prognostic indicator in the last decade. Although the exact mechanism that links tumor prognosis and NLR remains unclear, an increased NLR has been shown to have a more unsatisfactory outcome. A cutoff value of >5 has been consistently used. In a meta-analysis, Guthrie et al. noted that NLR is an independent prognostic factor in patients with solid organ malignancies in the upper gastrointestinal tract that tends to present at a later stage with more advanced features [21].

Gomez et al. reported that a preoperative NLR ≥ 5 indicates poor disease-free and overall survival following curative resection for HCC [22]. In addition, a high NLR was observed to significantly increase the risk of tumor recurrence and recipient mortality in patients who underwent LT for HCC [23,24,25,26]. In this study, NLR as a continuous variable showed significant prognostic value for HCCD; a higher NLR value combined with other tumor-specific factors showed an increased risk of HCC-specific death. 

Survival after LT for HCC is linked with tumor recurrence and the time of recurrence and duration of TBS. A longer TBS means more prolonged survival despite recurrence. In addition, early screening and aggressive local management of recurrence and the type and potency of immunosuppressants (incorporation of mTORi and level of calcineurin inhibitor) can also be associated with longer TBS. Therefore, these tumor-specific survival-related factors were collectively reflected in the SALT calculator. 

To provide reasonable explanations associated with different HCC-specific survival models, recurrence patterns and TBS were analyzed together with RFS between the different RS groups (Figure 3b and Table 3). Higher-mortality risk groups showed earlier HCC recurrence as well as a higher recurrence rate. Furthermore, higher-mortality risk groups showed a tendency for shorter TBS than low mortality risk groups. From this analysis, we learned that late recurrence and longer TBS could also be associated with more prolonged HCC-specific survival along with the recurrence rate itself. 

A debate is ongoing about the HCC recurrence or survival in the LDLT compared to DDLT. Our team also reported more unsatisfactory outcomes in LDLT compared to DDLT in a group of patients precisely following the UCSF criteria, especially in small living donor grafts [27]. Although, in general, studies showed higher recurrence in LDLT, the rates of HCC specific deaths have not been commonly evaluated between LDLT and DDLT groups. Therefore, we compared the outcomes between LDLT vs. DDLT among the patients with similar risks. There was no significant difference in recurrence and HCCD rates between LDLT and DDLT in all stratified risk groups. Based on this finding, we can confidently suggest that this model is applicable in both LDLT and DDLT cases. However, further investigations with a larger sample of both groups would still be needed. 

The limitation of this present study is the small number of data in the validation set. In addition, further analysis and multicenter validation set with more significant numbers of data should be performed to validate the SALT calculator further. 

## 5. Conclusions

This study proposes a new tailored predictor for tumor-specific survival after LT in HCC using preoperative parameters alone, named the SALT calculator. This calculator predicts HCC-specific survival accurately in internal and external validation. Thus, this calculator will be clinically helpful in predicting survival after LT for HCC preoperatively.

## Figures and Tables

**Table 1:** Demographic data of the derivation and validation set.

Variables		Values		
		Derivation Set	Validation Set	*p*-Value
Sex	Male Female	472 (81.7%) 106 (18.3%)	153 (87.4%) 22 (12.6%)	0.07
Age (year)	Mean ± SD	55.6 ± 8.3	56.7±6.3	0.06
Primary Disease	HBV HBV + HCV HCV NBNC Alcoholic Others	442 (76.5%) 16 (2.8%) 66 (11.4%) 18 (3.1%) 27 (4.7%) 9 (1.6%)	144 (82.3%) 4 (2.3%) 9 (5.1%) 6 (3.4%) 11 (6.3%) 1 (0.6%)	
MELD score	Median (range)	14.7 (0.9–49)	9 (5–40)	<0.01
Type of LT	DDLT LDLT	83 (14.4%) 495 (85.6%)	7 (4%) 168 (96%)	
AFP	Median (range)	9.6 (0.8–1,708,000)	7.2 (1.3–8367.7)	0.10
PIVKA-II	Mean ± SD (range)	29 (2–76,000)	27 (7–22,462)	0.80
NLR *	Median (range)	2.3 (0.4–92)	1.9 (0.6–34.9)	<0.01
PET	Positive Negative	212 (36.7%) 366 (63.3%)	72 (41.1%) 103 (58.9%)	0.28
Largest tumor size	Median (range)	26 (4–240)	25 (5–105)	0.16
Recurrence	Yes Intrahepatic Extrahepatic Both	102 (17.8%) 34 (5.9%) 25 (4.3%) 43 (7.4%)	52 (29.7%) 23 (13.1%) 15 (8.6%) 14 (8%)	
Follow-up Period (months)	Median (range)	65.5 (0–154)	34 (0–71)	

* NLR is defined as relative (%) neutrophil/relative (%) lymphocyte; SD, standard deviation; HBV, hepatitis B virus; HCV, hepatitis C virus; MELD, model of end-stage liver disease; NBNC, non-B non-C hepatocellular carcinoma; LDLT, living donor liver transplantation; DDLT, deceased donor liver transplantation; AFP, alpha-fetoprotein; PIVKAII, protein induced by vitamin K absence/antagonist-II; NLR, neutrophil-to-lymphocyte ratio; PET, positron emission tomography.

**Table 2:** Risk factors for HCC-specific deaths.

Variable	Multivariable Analysis	
	Shr * (95% CI)	*p*-Value
Sex		
Female	Reference	
Male	2.61 (1.21–5.63)	0.01
Largest tumor size	1.01 (1–1.01)	0.04
PET		
Negative	Reference	
Positive	2.41 (1.46–3.97)	<0.01
Ln(AFP+1)	1.24 (1.12–1.38)	<0.01
Ln(PIVKA-II)	1.18 (1.03–1.35)	0.01
NLR	1.02 (1.01–1.04)	<0.01

* sHR, sub-distribution hazard ratio. CI, confidence interval; NLR, neutrophil-to-lymphocyte ratio; AFP, alpha-fetoprotein; PIVKAII, protein induced by vitamin K absence/antagonist-II.

**Table 3:** Uno’s c-Index for the internal and external validation of the developed model.

**Uno’s c-Index** **(95% CI)**	Model	0.8 (0.75, 0.86)
	Internal *	0.79
	external	0.75 (0.65, 0.86)
**Calibration slope** **(95% CI)**	Model	1 (0.84, 1.16)
	Internal *	0.85
	external	0.9 (0.49, 1.3)

* bootstrap validation with 1000 bootstrap samples.

**Table 4:** Cumulative incidence rates of HCC-specific death based on range of risk score (RS).

	1 Year	3 Years	5 Years	
Group I (*n* = 74) (RS ≤ 1.91)	0	0	2% (0.1–7.7)	
Group II (*n* = 433) (RS 1.91–4.07)	1% (0.4–2.6)	7.7% (5.4–10.5)%	11% (8.3–14.6)	
Group III (*n* = 47) (RS > 4.07)	32% (19.1–45.5)	64% (47.7–76.9)	64% (47.7–76.9)	*p* < 0.01 *

* Gray’s test.

**Table 5:** Cumulative incidence rates of HCC recurrence based on range of risk score (RS).

	1 Year	3 Years	5 Years	
Group I (*n* = 74) (RS ≤ 1.91)	0	0	5%	
Group II (*n* = 433) (RS 1.91–4.07)	5%	12.5%	14%	
Group III (*n* = 47) (RS > 4.07)	56%	78%	82%	*p* < 0.01 *

* Log-Rank test.

**Table 6:** Real cumulative incidence of mortality in derivation set based on range of risk score (RS).

	1 Year	3 Years	5 Years	
Group I (*n* = 74) (RS ≤ 1.91)	0	0	2%	
Group II (*n* = 433) (RS 1.91–4.07)	3%	10%	14%	
Group III (*n* = 47) (RS >4.07)	41%	76%	76%	*p* < 0.01 *

* Log-Rank test, * Risk score (RS) calculated as 0.96×Sex(M)+0.01×biggest(mm)+0.88×pet(+)+0.21×ln(AFP+1)+0.17×ln (PIVKAII)+0.02×NLR.

**Table 7:** Recurrence rate and recurrence pattern in three groups based on risk score RS* and comparison between groups.

Groups	Recurrence	Initial Sites of Recurrence					Recurrence-to-Death Median (Range) **
		Intrahepatic **	Extrahepatic **			Combined **	
			Lung	Bone	Others		
Group I (*n* = 74) (RS ≤ 1.91)	3 (4.1%)	0	1 (33.3%)	0	0	2 (66.7%)	78 (0–98)
Group II (*n* = 433) (RS 1.91–4.07)	62 (14.3%)	21 (33.9%)	4 (6.5%)	3 (4.8%)	1 (1.6%)	33 (53.2%)	6.5 (0–136) †
Group III (*n* = 47) (RS > 4.07)	34 (72.3%)	16 (47.1%)	3 (8.8%)	2 (5.9%)	1 (2.9%)	12 (35.3%)	2.5 (0–44) †
Total	99 (17.9%)	37 (37.4%)	8 (8.1%)	5 (5.1%)	2 (2%)	47 (47.5%)	15.20 ± 23.02 (0–136)

RS was not calculated in 24 cases with no NLR values, ** Calculated in recurred cases, † Group II vs. Group III (*p* = 0.06).

## Data Availability

Research data are not shared.

## Abbreviations

[18]-FDG PET	[18]F-fluorodeoxyglucose positron emission tomography
AFP	alpha-fetoprotein; CT: computed tomography
DDLT	deceased donor liver transplantation
HCC	hepatocellular carcinoma
HCCD	cumulative incidence of HCC-specific death
IP	incidence probability
LDLT	living donor liver transplant
LT	liver transplantation
MoRAL	model to predict tumor recurrence after LDLT
MRI	magnetic resonance imaging
mTORi	mammalian target of rapamycin inhibitors
NBNC	non-B non-C hepatocellular carcinoma
NLR	neutrophil-to-lymphocyte ratio
PET	positron emission tomography
PIVKAII	protein induced by vitamin K absence/antagonist-II
RS	risk score
SALT	survival after liver transplantation for HCC
SNUH	Seoul National University Hospital
TBS	tumor-bearing survival
UCSF	University of California, San Francisco
